# A Theoretical Study on Laser Cooling Feasibility of Group IVA Hydrides XH (X = Si, Ge, Sn, and Pb): The Role of Electronic State Crossing

**DOI:** 10.3389/fchem.2020.00020

**Published:** 2020-01-28

**Authors:** Donghui Li, Mingkai Fu, Haitao Ma, Wensheng Bian, Zheng Du, Congmei Chen

**Affiliations:** ^1^Beijing National Laboratory for Molecular Sciences, Institute of Chemistry, Chinese Academy of Sciences, Beijing, China; ^2^School of Chemical Sciences, University of Chinese Academy of Sciences, Beijing, China; ^3^Institute of Electrical Engineering, Chinese Academy of Sciences, Beijing, China; ^4^National Supercomputing Center in Shenzhen, Shenzhen University Town, Shenzhen, China

**Keywords:** laser cooling, *ab initio*, spin-orbit coupling, electronic state crossing, group IVA hydrides

## Abstract

The feasibility of direct laser cooling of SiH, GeH, SnH, and PbH is investigated and assessed based upon first principles. The internally contracted multi-reference configuration interaction method with the Davidson correction is applied. Very good agreement is obtained between our computed spectroscopic constants and the available experimental data. We find that the locations of crossing point between the B^2^Σ^−^ and A^2^Δ states have the tendency of moving downwards from CH to SnH relative to the bottom of the corresponding A^2^Δ potential, which precludes the laser cooling of GeH, SnH, and PbH. By including the spin-orbit coupling effects and on the basis of the ${\text{A}}^{2}\Delta_{5/2}\to$${\text{X}}^{2}\Pi_{3/2}$ transition, we propose a feasible laser cooling scheme for SiH using three lasers with wavelengths varying from 400 to 500 nm, which features a very large vibrational branching ratio (0.9954) and a very short radiative lifetime (575 ns). Moreover, similar studies are extended to carbon monosulfide (CS) with a feasible laser cooling scheme proposed. The importance of electronic state crossing in molecular laser cooling is underscored, and our work suggests useful caveats to the choice of promising candidates for producing ultracold molecules.

## 1. Introduction

Laser cooling of diatomic molecule is an issue of great interest owing to their promising applications in many fields such as quantum computing and precision measurement (Carr et al., 2009; Hudson et al., 2011; Yan et al., 2013; Baron et al., 2014). Direct laser cooling of SrF molecules to μK level was firstly achieved with only three laser beams (Shuman et al., 2010). In addition, transverse and longitudinal laser cooling experiment was applied to YO (Hummon et al., 2013) and CaF (Zhelyazkova et al., 2014), respectively. So far only a few molecules including SrF, CaF, and YO have been cooled to the ultracold temperature experimentally, and there is an urgent need to search for more suitable molecular candidates for laser cooling. Recently, a number of theoretical efforts have been made in searching for promising molecular laser cooling candidates (Wells and Lane, 2011a; Fu et al., 2016a; Cao et al., 2019; González-Sánchez et al., 2019; Xu et al., 2019). It is generally accepted (Di Rosa, 2004; Fu et al., 2016b) that, a suitable laser-cooling candidate must meet the following three criteria: highly diagonal Franck-Condon factors (FCFs), a short lifetime, and no intermediate electronic-state interference. Here we reveal that the crossing between two electronic states may damage the cooling scheme, and may be regarded as the fourth criterion that should be checked beforehand in choosing laser-cooling candidates. We will demonstrate this point by investigating the laser cooling of group IVA hydrides.

There have been a lot of studies on SiH and GeH (Kleman and Werhagen, 1953a; Ram et al., 1998), while SnH and PbH have not been sufficiently studied (Alekseyev et al., 1996; Zhao et al., 2017). SiH radical has been attracting great interest over many years, since it plays a significant role in many industrial processes such as plasma vapor deposition (Ram et al., 1998). In 1930, the emission spectrum of SiH was observed using an arc source, and a strong transition near 410 nm was assigned to as the A^2^Δ→X^2^Π transition (Jackson, 1930). In 1969, the lifetime of the A^2^Δ state was measured as 700±100 ns with phase-shift technique (Smith, 1969). Later, the high-resolution spectrograms of 325 nm bands for SiH were measured with rotational analysis (Bollmark et al., 1971). In 1979, the accurate spectroscopic parameters and molecular constants of SiH were summarized and reported in the literature (Huber and Herzberg, 1979). In 1980, the emission spectroscopy of SiH in a silane glow-discharge was measured with a moderate spectrometer (Perrin and Delafosse, 1980). In 1989, the spectrum of SiH radical was observed over the laser wavelength between 426 and 430 nm by resonance-enhanced multiphoton ionization spectroscopy, and new state was tentatively assigned as ^2^Π state with further computer simulation (Johnson and Hudgens, 1989). Parallel to extensive studies on SiH, the spectra of GeH are well-studied in experiment, especially for the low-lying electronic states. In 1953, the spectra of A^4^Σ^−^→X^2^Π and A^2^Δ→X^2^Π band for GeH were first observed (Kleman and Werhagen, 1953a,b). Subsequently, the spectroscopic constants of the A^2^Δ state of GeH were deduced with rotational analysis (Klynning and Lindgren, 1966). In 1989, the radiative lifetime of the A^2^Δ state of GeH was measured as 93±10 ns with laser-induced fluorescence technique (Bauer et al., 1989). In 1938, the emission spectrum of PbH was firstly detected using arc-excitation in hydrogen atmosphere (Watson, 1938). In 2008, the near-infrared emission spectra of ${\text{X}}^{2}\Pi_{3/2}\to^{2}\Pi_{1/2}$ transitions of PbH were observed using high-resolution Fourier-transform spectrometry (Setzer et al., 2008).

Theoretically, in 1967 the first *ab initio* calculations on the SiH radical were performed with the Hartree-Fock method (Cade and Huo, 1967), in which the potential energy curve (PEC) of the ground-state was obtained. In 1983, the PECs of SiH were evaluated with the multi-reference double-excitation configuration interaction calculations (Lewerenz et al., 1983). In recent years, there are many calculations about spectroscopic constants of SiH (Ram et al., 1998; Kalemos et al., 2002; Shi et al., 2008, 2013; Zhang et al., 2018). In 2013, the PECs of eleven Ω states generated from seven Λ-S states of SiH radical were calculated by the internally contracted multi-reference configuration interaction method with the Davidson correction (icMRCI+Q) (Shi et al., 2013). In 2001, the X^2^Π and a^4^Σ^−^ states of GeH were investigated with the MRCI approach and the spectroscopic parameters for the two states were reported (Bruna and Grein, 2001). In 2015, the lifetimes of A^2^Δ and a^4^Σ^−^ states of GeH were obtained (Li et al., 2015). Some *ab initio* calculations on SnH and PbH were also reported (Alekseyev et al., 1996; Zhao et al., 2017).

The A^2^Δ→X^2^Π transition of CH was pointed out to be dominated by diagonal bands, making it a potential cooling candidate (Wells and Lane, 2011b). SiH is considered to be suitable for laser cooling by Zhang et al. (2018), although they ignored the SOC effects in their laser cooling schemes. In addition, they did not consider the effect of electronic state crossing. According to previous investigations (Fu et al., 2017; Xia et al., 2017), the SOC effects are important for laser cooling molecules and will be taken into account in this work. There have not been theoretical studies reported on laser cooling of GeH, SnH, and PbH. In this work, by means of highly accurate *ab initio* and dynamical calculations, we investigate the laser cooling of group IVA hydrides and the importance of electronic state crossing in molecular laser cooling is underscored. Additionally, based on the fourth criterion mentioned above, laser cooling of CS is investigated.

The paper is organized as follows. The theoretical methods and computational details are briefly described in section 2. The results and discussion are shown in section 3. The conclusions are given in section 4.

## 2. Methods and Computational Details

In this paper, all the *ab initio* calculations of SiH, GeH, SnH, and PbH are performed using the MOLPRO 2012.1 program package (Werner et al., 2012). For each Λ-S state, the energies are computed using the complete active space self-consistent field (CASSCF) (Werner and Knowles, 1985) approach followed by the icMRCI+Q (Langhoff and Davidson, 1974). The lowest electronic configuration of Si is (core)3s^2^3p^2^ corresponding to the atomic states ^3^*P* and ^1^*D*. In combination with the 1 s function of a H atom, the lowest electronic configuration of SiH in X^2^Π is invariably (core)4σ^2^5σ^2^6σ^0^2π^1^ and the next is (core)4σ^2^5σ^1^6σ^0^2π^2^ leading to the multiplets ^4^Σ^−^, ^2^Δ, ^2^Σ^−^, and ^2^Σ^+^, which could be considered as involving a *pσ*→*pπ* transition within the Si atom. In asymptote region, the 5σ molecular orbital origins from the 1s on hydrogen, while the 6σ and 2π correspond to the 3*p* on silicon.

The choice of active space is very important in the CASSCF and MRCI+Q calculations (Liu et al., 2009; Yu and Bian, 2011, 2012). The active space of SiH used here consists of ten orbitals corresponding to Si 2p3s3p4p_π_ and H 1s, and is referred to as (11e, 10o), which is carefully chosen to include proper orbitals, and large enough for the present calculations. Our test calculations indicate that the full valence space is inadequate, and thus additional five orbitals are added into the active space. The inner shell orbitals are included to account for the core-valence correlation effects and the outer virtual orbitals are added to give a better description on the dissociation behavior and Rydberg character, especially for excited electronic states (Shen et al., 2017). In fact, a completely satisfactory set could neither be found by increasing the active orbital space nor by including more states (Simah et al., 1999). The best compromise we could achieve is to distribute the eleven electrons in 10 active orbitals. As for the basis set, we use the aug-cc-pV5Z basis sets for Si and H atom. In the SOC calculations, the SO eigenstates are obtained by diagonalizing Ĥ^*el*^+Ĥ^*SO*^ in the basis of eigenfunctions of Ĥ^*el*^. In addition, the Ĥ^*el*^ and Ĥ^*SO*^ are obtained from the icMRCI+Q calculations and icMRCI+Q waves functions, respectively. The active space for GeH in the present work is (9e, 9o), which consists of the Ge 3d_π_4s4p5p_π_ and H 1s orbitals. The accuracy and computational performance are compromised, thus the active space of GeH includes the 3d_π_ orbitals, valence orbitals and 5p_π_ orbitals. The aug-cc-pV5Z-DK basis sets are used for GeH. Here, the active space for SnH consists of the Sn 4d5s5p and H 1s orbitals and is referred as (15e, 10o). The aug-cc-pVQZ basis sets are used for H and aug-cc-pwCVQZ-PP with ECP28MDF effective core potentials for Sn. Similarly, the active space for PbH is (15e, 10o), which consists of the Pb 5d6s6p and H 1s orbitals. We use the aug-cc-pVQZ basis sets for H and aug-cc-pwCVQZ-PP with ECP60MDF for Pb. In addition, the active space used for the study of CS is (10e, 8o) corresponding to the C 2s2p and S 3s3p, and we use the aug-cc-pV5Z basis sets for both C and S atoms.

The Einstein spontaneous emission coefficient $A_{\nu^{\prime }\nu }$ is calculated by the following expression (Herzberg, 1950),

(1) $$\begin{array}{l} A_{\nu^{\prime }\nu }=\frac{16\pi^{3}}{3\epsilon_{0}\hbar }\frac{S\left(J^{\prime },J\right)}{2J^{\prime }+1}v^{3}|{\text{Ψ}}_{\nu^{\prime },J^{\prime }}|M\left(r\right)|{\text{Ψ}}_{\nu ,J}{|}^{2} \end{array}$$

where $A_{\nu^{\prime }\nu }$ is in units of *s*^−1^, ε_0_ is the vacuum permittivity in units of F· cm^−1^, *M*(*r*) is the transition dipole function in Debye unit, ν is emission frequency in units of cm^−1^, ${\text{Ψ}}_{\nu^{\prime },J^{\prime }}$ and Ψ_ν, *J*_ are normalized radial wave functions, ℏ is the Planck constant and *S*(*J*′, *J*) is the Hönl-London rotational intensity factor. The radiative lifetime for a given vibrational level ν′ can be obtained by the following equation,

(2) $$\begin{array}{l} \tau_{\nu^{\prime }\nu }=1/\underset{\nu }{\sum }A_{\nu^{\prime }\nu } \end{array}$$

The spectroscopic parameters of group IVA hydrides, including the equilibrium bond length (*R*_*e*_), the harmonic vibrational constant (ω_*e*_), the rotational constant (*B*_*e*_), and adiabatic relative electronic energy referred to the ground state (*T*_*e*_) are obtained using the Le Royν′s LEVEL program (Le Roy, 2007).

## 3. Results and Discussion

### 3.1. PECs of the Λ-S States and Spectroscopic Constants

**Figure 1:**
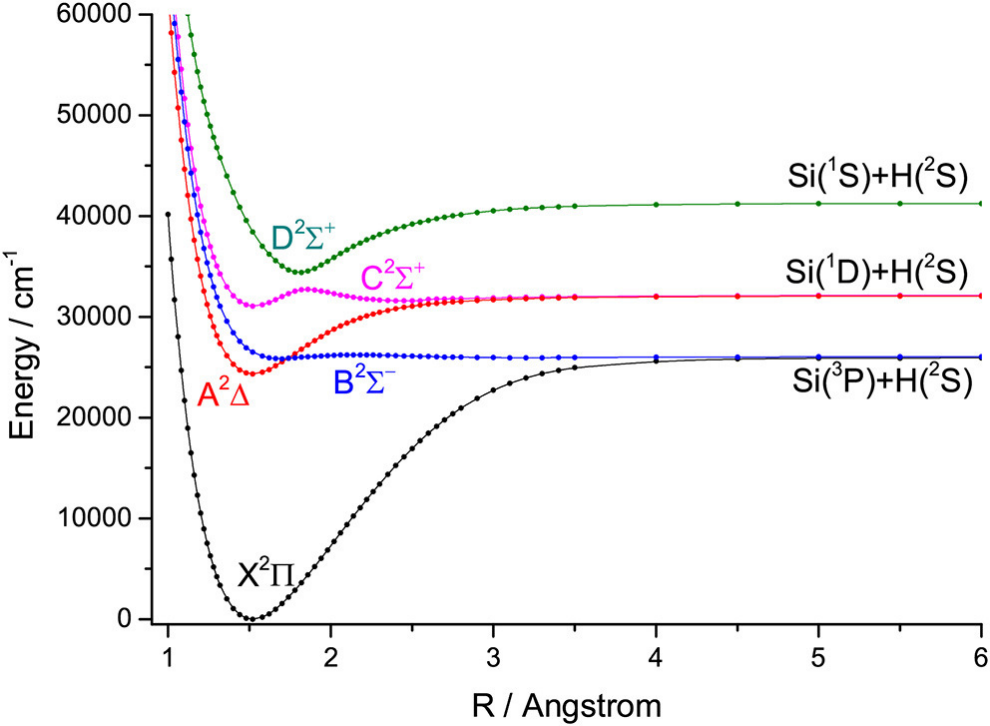
Potential energy curves of SiH for the five Λ-S states at the icMRCI+Q level.

**Figure 2:**
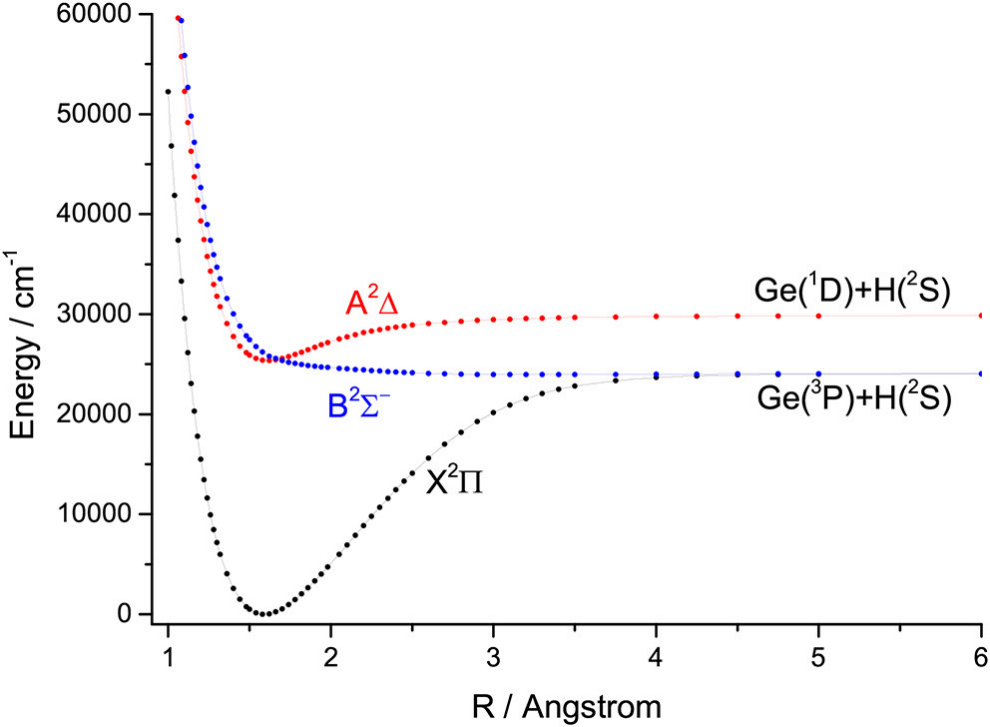
Potential energy curves of GeH for the three Λ-S states at the icMRCI+Q level.

**Figure 3:**
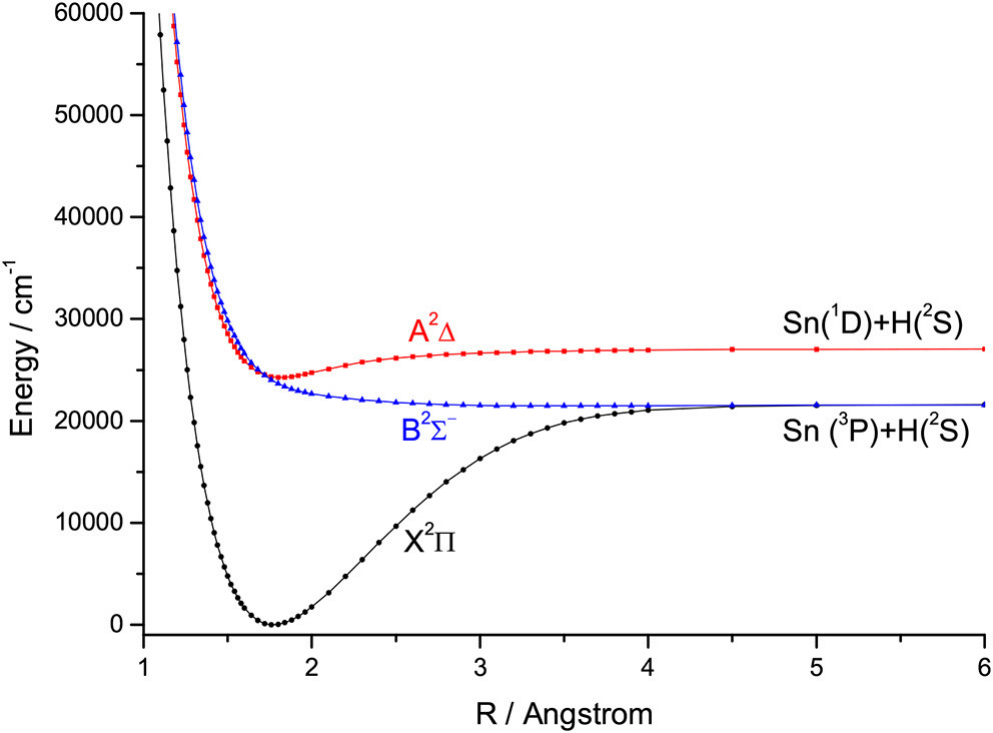
Potential energy curves of SnH for the three Λ-S states at the icMRCI+Q level.

**Figure 4:**
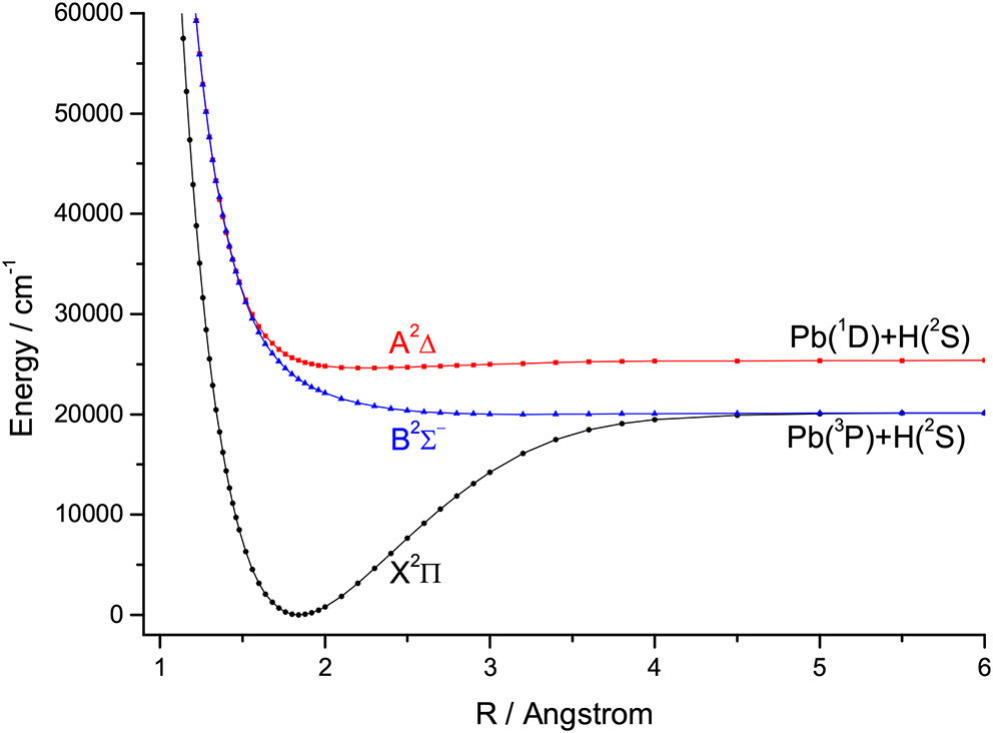
Potential energy curves of PbH for the three Λ-S states at the icMRCI+Q level.

For the group IVA hydrides, the main configuration of the ground state (X^2^Π) is (core)4σ^2^5σ^2^6σ^0^2π^1^. The second lowest state here is the A^2^Δ, whose main configuration is (core)4σ^2^5σ^1^6σ^0^2π^2^. The PECs of SiH, GeH, SnH, and PbH computed using the icMRCI+Q method are shown in Figures 1–4, respectively. The weights of ionic configuration in the X^2^Π and A^2^Δ states decrease gradually with increasing internuclear distances. As seen in Figure 1, the five states of SiH correlate to the three dissociation limits. The X^2^Π and A^2^Δ states are the two energetically lowest lying electronic state and correlate to the neutral atomic Si(^3^P)+H(^2^S) limit and Si(^1^D)+H(^2^S) limit, respectively. From Figures 2–4, we can see that the X^2^Π and A^2^Δ states of GeH, SnH, and PbH correlate to the neutral atomic Ge/Sn/Pb(^3^P)+H(^2^S) limit and Ge/Sn/Pb(^1^D)+H(^2^S) limit, respectively. The PEC of the B^2^Σ^−^ crosses with that of A^2^Δ will be discussed in details in section 3.2. The PECs of the B^2^Σ^−^ and X^2^Π correlate to the same neutral atomic Si(^3^P)+H(^2^S) limit.

Since the spectroscopic constants of the X^2^Π and A^2^Δ states have been measured in experiment for SiH, GeH, SnH, and PbH, comparing with experimental data could provide an indicator of the reliability of our calculations. We present our calculated spectroscopic constants of SiH, GeH, and SnH in Tables 1, 2 and Table S1, together with previous theoretical and experimental values for comparison. The calculated *R*_*e*_ and ω_*e*_ of the X^2^Π of SiH are 1.5200 Å and 2047.71 cm^−1^, respectively, which are in good agreement with the corresponding experiment data (1.5201 Å and 2041.80 cm^−1^) (Huber and Herzberg, 1979). The contributions from non-valence orbitals to correlation energy are relatively small for the ground state, however, they become very important for the excited states and lead to noticeable differences. In particular, the experimental *T*_*e*_ is 24300.4 cm^−1^ (Huber and Herzberg, 1979) and our calculated one with a better active space is 24299.20 cm^−1^, whereas that obtained by Zhang et al. (2018) is 25080.92 cm^−1^, which deviates from experimental value by about 780 cm^−1^. When additional virtual orbitals are taken into account by Kalemos et al. (2002), the deviation is reduced to around 200 cm^−1^. The spectroscopic constants of GeH obtained from the active space (9e, 9o) are shown in Table 2. The calculated *R*_*e*_ and ω_*e*_ of the X^2^Π of GeH are 1.5885 Å and 1902.32 cm^−1^, respectively, which are in very good agreement with the corresponding experiment data (1.5872 Å and 1900.38 cm^−1^) (Towle and Brown, 1993). The A^2^Δ state of GeH, the experimental *T*_*e*_ is 25454 cm^−1^ (Huber and Herzberg, 1979), whereas our calculated *T*_*e*_ is 25386.63 cm^−1^, which is much improvement compared with previous computational value 25774 cm^−1^ (Li et al., 2015).

**Table 1:** Spectroscopic constants of the A^2^Δ and X^2^Π states for SiH.

**State**	**Method**	**T_*e*_**	**R_*e*_**	**ω_*e*_**	**ν^a^**	**T_**ν**_**	**B_*e*_**	**μ_*e*_**
		**(*cm^**−1**^*)**	**(Å)**	**(*cm^**−1**^*)**		**(*****cm^**−1**^*)**	**(*****cm^**−1**^*)**	**(Debye)**
X^2^Π	This work		1.5200	2047.71	0	1018.07	7.5083	0.1451
					1	2996.15		
					2	4904.97		
	Expt.^b^		1.5201	2041.80			7.4996	
	Expt.^c^		1.5197	2042.52			7.5039	
	Calc.^d^		1.5223	2043.15				
	Calc.^e^		1.5223	2046.91			7.4779	
	Calc.^f^		1.5154	2043.47			7.7258	
A^2^Δ	This work	24299.20	1.5238	1859.72	0	25277.40	7.5164	0.134
					1	26988.69		
					2	28485.89		
	Expt.^b^	24300.4	1.5234	1858.90			7.4664	
	Expt.^g^	24255.51	1.5198				7.5027	
	Calc.^h^	24923.56	1.546	1797			7.253	0.118
	Calc.^d^	24129.60	1.5240	1853.15				
	Calc.^f^	24323.28	1.5148	1857.63			7.5021	

a *The vibrational levels are represented by ν*.

b *Huber and Herzberg (1979)*.

c *Betrencourt et al. (1986)*.

d *Kalemos et al. (2002)*.

e *Shi et al. (2008)*.

f *Shi et al. (2013)*.

g *Ram et al. (1998)*.

h *Lewerenz et al. (1983)*.

**Table 2:** Spectroscopic constants of the A^2^Δ and X^2^Π states for GeH.

**State**	**Method**	**T_*e*_**	**R_*e*_**	****ω_*e*_****	****ν^a^****	**T_**ν**_**	**B_*e*_**	**μ_*e*_**
		**(*cm^**−1**^*)**	**(Å)**	**(*cm^**−1**^*)**		**(*cm^**−1**^*)**	**(*cm^**−1**^*)**	**(Debye)**
X^2^Π	This work		1.5885	1902.32	0	943.46	6.7107	0.1076
					1	2777.80		
					2	4546.19		
	Expt.^b^		1.5872	1900.38			6.73	
	Expt.^c^		1.5880	1833.77			6.7259	
	Calc.^d^							0.097
	Calc.^e^		1.5823	1914.93			6.7688	
A^2^Δ	This work	25386.63	1.6100	1308.62	0	26159.92	6.5408	0.257
					1	27380.21		
					2	28307.39		
	Expt.^c^	25454	1.611	1185.15			6.535	
	Calc.^d^	26663	1.66	1302				
	Calc.^e^	25774	1.617	1306.36			6.5343	0.356

a *The vibrational levels are represented by ν*.

b *Towle and Brown (1993)*.

c *Huber and Herzberg (1979)*.

d *Chapman et al. (1988)*.

e *Li et al. (2015)*.

**Figure 5:**
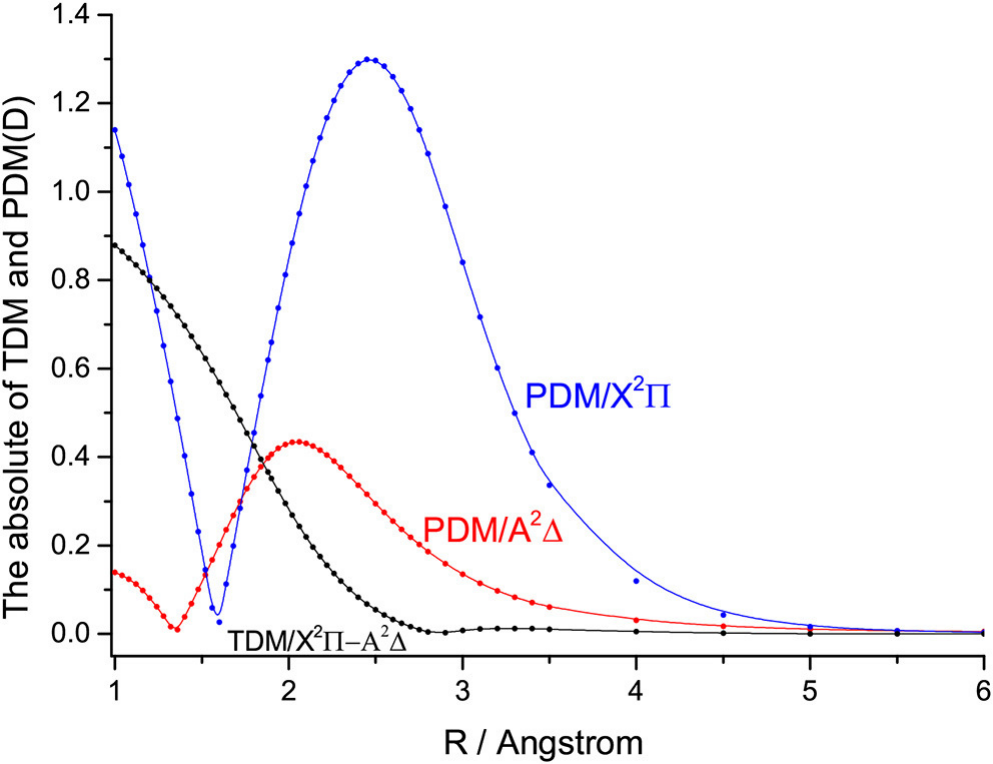
The permanent dipole moments (PDMs) and transition dipole moments (TDMs) for the X^2^Π and A^2^Δ states of SiH at the icMRCI+Q level.

The permanent dipole moments (PDMs) and absolute values transition dipole moments (TDMs) for the A^2^Δ→X^2^Π transition of SiH at the icMRCI+Q level are represented in Figure 5. The TDMs for the A^2^Δ→X^2^Π transition of SiH are 0.6231 D at *R*_*e*_ and decrease with the increasing bond length. The calculated TDMs for the A^2^Δ→X^2^Π transition of GeH are given in Figure S1. The Einstein A coefficients and vibrational branching ratio of the A^2^Δ→X^2^Π transition of GeH are listed in Table S2. The calculated radiative lifetime for the A^2^Δ(ν′ = 0) state of SiH is 613 ns, which is in very good agreement with the experimental value (534 ± 23 ns) (Bauer et al., 1984). The FCFs $f_{\nu^{\prime }\nu }$ of the A^2^Δ→X^2^Π transition for GeH are calculated and plotted in Figure S2. In this work, the *f*_00_ (0.995) of SiH is in excellent agreement with the value (0.994) derived from experiment (Smith and Liszt, 1971). The computational value of *f*_00_ for GeH (0.940) is in very good agreement with the experimental value 0.928 (Erman et al., 1983).

### 3.2. The Comparison of the Feasibility of Laser Cooling for Group IVA Hydrides

**Figure 6:**
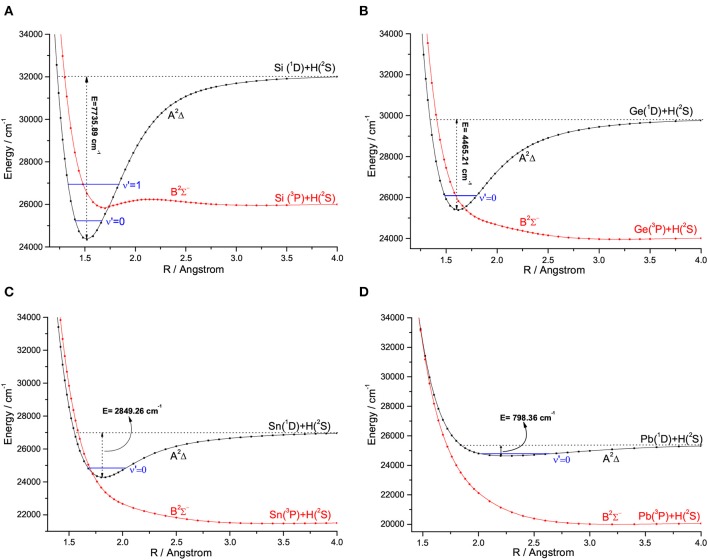
An amplified view of crossing regions of potential energy curves for SiH **(A)**, GeH **(B)**, SnH **(C)**, and PbH **(D)**.

There are some similar properties of group IVA hydrides. The inner closed-shell orbitals are occupied with 2, 10, 28, 46, and 78 electrons for CH, SiH, GeH, SnH, and PbH, respectively. The equilibrium bond length *R*_*e*_ increases regularly from CH to PbH, while the harmonic constant ω_*e*_ decreases in the order of CH>SiH>GeH>SnH>PbH (Huber and Herzberg, 1979; Alekseyev et al., 1996; Zhao et al., 2017). The comparison of the feasibility of laser cooling for group IVA hydrides is summarized here. An amplified view of crossing regions of PECs for SiH, GeH, SnH, and PbH is shown in Figure 6, we see that the dissociation energies of the A^2^Δ states of SiH, GeH, SnH, and PbH are 7735.89, 4465.21, 2849.26, and 798.36 cm^−1^, respectively. This trend is consistent when the second-row CH is included and the corresponding dissociation energy is 16641.68 cm^−1^ (Wells and Lane, 2011b). The depths of the A^2^Δ state decrease from CH to PbH, and it supports only one vibrational level for PbH. In equilibrium region, two electrons anti-parallelly are distributed on one (n$p_{x}^{↑↓}$/n$p_{y}^{↑↓}$) and two (n$p_{x}^{↑}np_{y}^{↓}$) sp^3^ hybridized orbitals for the B^2^Σ^−^ and A^2^Δ states, respectively. The hybridized orbital effect vanishes as the internuclear distance increases to the asymptotic region. Where, n$p_{x}^{↑↓}$/n$p_{y}^{↑↓}$ goes to X(^3^P) and n$p_{x}^{↑}np_{y}^{↓}$ goes to X(^1^D). Therefore, there is a crossing point between the B^2^Σ^−^ and A^2^Δ states of group IVA hydrides. The electronic state crossing between the B^2^Σ^−^ and A^2^Δ states can lead to nonradiative transition (Wu et al., 2019), and may cause predissociation. This kind of electronic state crossing in a diatomic molecule will become potential energy surface intersections in the polyatomic cases involving multiple electronic states (Liu et al., 2003; Zhao et al., 2006). We find that the locations of crossing point between the B^2^Σ^−^ and A^2^Δ states have the tendency of moving downwards from CH to SnH relative to the bottom of the corresponding A^2^Δ state potential. The locations of crossing point between the B^2^Σ^−^ and A^2^Δ states of GeH and SnH are 591 and 255 cm^−1^ lower than the corresponding vibrational level ν′ = 0 in the A^2^Δ, while that of third-row SiH is 670 cm^−1^ higher than the vibrational level ν′ = 0 in the A^2^Δ. This trend is consistent when the second-row CH is included since the corresponding crossing point is 3,000 cm^−1^ higher (Wells and Lane, 2011b).

The location of crossing point between the B^2^Σ^−^ and A^2^Δ states of SiH is higher than the vibrational level ν′ = 0 in the A^2^Δ, indicating that laser cooling of SiH in the A^2^Δ→X^2^Π transition may not be affected by electronic state crossing. However, our results imply that the crossing between the B^2^Σ^−^ and A^2^Δ states of GeH will lead to predissociation of all vibrational levels of the A^2^Δ state, which is backed up by experiments of Erman et al. (1983) using high frequency deflection technique. They reported that, 80–90% of the GeH molecules excited to the A^2^Δ state decay via predissociation to their ground state atomic constituents, and fewer than 20% of the molecules follow the regular decay route to the ground state. Furthermore, the A^2^Δ states of SnH and PbH have a similar problem to that of GeH, although there has been no relevant experimental measurements reported. It is clear that GeH and SnH can not be used for laser cooling due to the electronic state crossing. In addition, the small Franck-Condon factor *f*_00_ (0.08) of PbH also suggests that it is not suitable for laser cooling.

It seems that both CH and SiH are very good laser cooling candidates, and we make a comparison in the following. The *f*_00_ of SiH (0.995) is close to that of CH (0.9957) (Wells and Lane, 2011b). The radiative lifetimes of the A^2^Δ state of SiH and CH are 575 and 536 ns (Wells and Lane, 2011b), respectively. The T_*Doppler*_ and T_*recoil*_ of SiH (6.65 and 3.89 μK) are also similar to that of CH (7.13 and 7.91 μK) (Wells and Lane, 2011b). The calculated pump and repump wavelengths for SiH and CH are all in the visible region. The electronic state crossing between the B^2^Σ^−^ and A^2^Δ states of CH is higher than that of SiH, with both crossing points located above the corresponding vibrational ν′ = 0 levels in the A^2^Δ state. Generally speaking, a larger atomic mass difference for the diatomic candidate is desirable by experimentalists, and in this respect, SiH is better than CH. Furthermore, we will propose a scheme using two spin-orbit states for SiH in the next section, which is more feasible than the one using two Λ-S states for CH. The A^2^Δ→X^2^Π transition of CH was used to establish a laser cooling scheme, and the SOC effects were not included (Wells and Lane, 2011a). With the inclusion of the SOC effects into the icMRCI wave functions in our calculations, accurate Ω states are determined. The calculated spectroscopic constants of SiH are in very good accordance with experimental measurements. The prospect for the production of ultracold SiH molecules by means of direct laser cooling method is discussed below.

### 3.3. Laser Cooling Scheme for SiH

**Figure 7:**
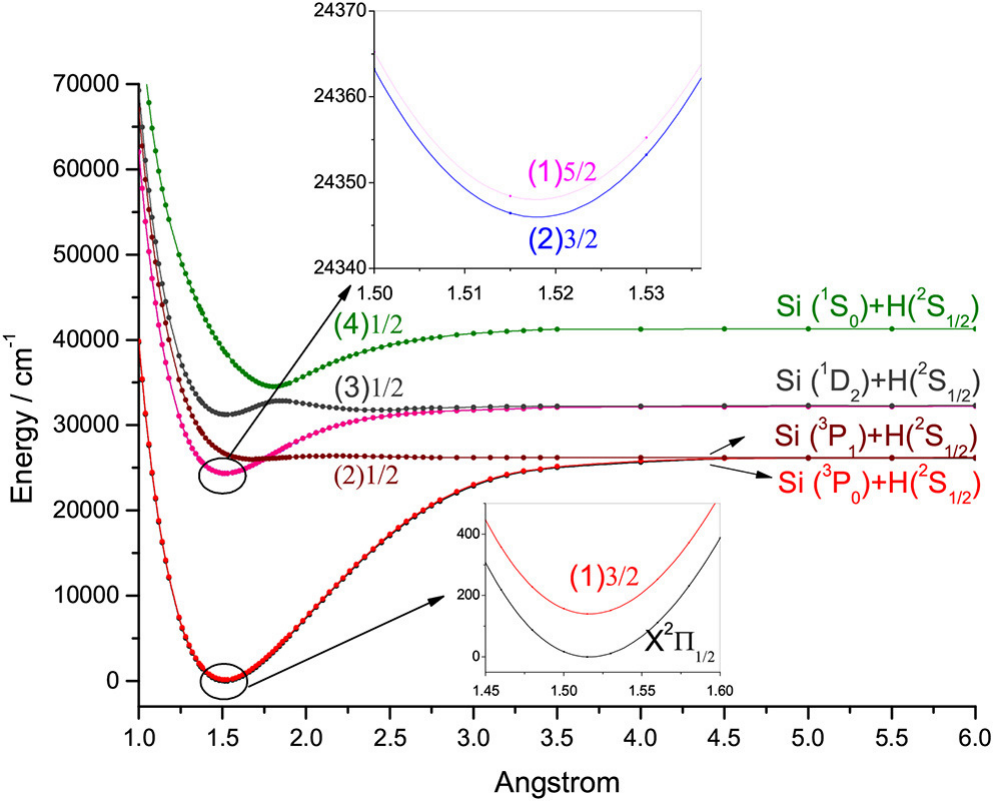
Potential energy curves of SiH for the seven Ω states at the icMRCI+Q level.

When the SOC effects are taken into account, seven Ω states, involving four states with Ω = 1/2, two states with Ω = 3/2 and one state with Ω = 5/2, are generated from the five Λ-S states of SiH. The PECs of the Ω states are depicted in Figure 7. The SOC splitting of the X^2^Π and A^2^Δ states of SiH is shown in Table 3. As seen, the energy separation of the ${\text{X}}^{2}\Pi_{1/2}$ and ${\text{X}}^{2}\Pi_{3/2}$ is 140.18 cm^−1^ in this work, which is in excellent agreement with the experimental value (142.83 cm^−1^) (Huber and Herzberg, 1979). For the ${\text{A}}^{2}\Delta_{3/2}$ and ${\text{A}}^{2}\Delta_{5/2}$ states, the energy separation is 3.01 cm^−1^.

**Table 3:** The spin-orbit coupling (SOC) splitting for SiH.

**State**	**Method**	**SOC splitting(*cm^**−1**^*)**
X1/2, (1)3/2	This work	140.18
	Expt.^a^	142.83
	Calc.^b^	141.12
	Calc.^c^	118.5
	Calc.^d^	141.0343
(2)3/2, (1)5/2	This work	3.01
	Expt.^a^	3.58
	Calc.^b^	5.19
	Calc.^c^	0.1
	Calc.^d^	2.7135

a *Huber and Herzberg (1979)*.

b *Shi et al. (2013)*.

c *Li et al. (2008)*.

d *Zhang et al. (2018)*.

We find that, the ${\text{A}}^{2}\Delta_{5/2}$→${\text{X}}^{2}\Pi_{3/2}$ transition is suitable for laser cooling in three possible transitions, ${\text{A}}^{2}\Delta_{3/2}$→${\text{X}}^{2}\Pi_{1/2}$, ${\text{A}}^{2}\Delta_{3/2}$→${\text{X}}^{2}\Pi_{3/2}$, and ${\text{A}}^{2}\Delta_{5/2}$→${\text{X}}^{2}\Pi_{3/2}$. In the ${\text{A}}^{2}\Delta_{5/2}$→${\text{X}}^{2}\Pi_{3/2}$ transition, SiH molecules will jump from the ${\text{X}}^{2}\Pi_{3/2}$(ν = 0) to the ${\text{A}}^{2}\Delta_{5/2}$(ν′ = 0), then the ${\text{A}}^{2}\Delta_{5/2}$(ν′ = 0) state will decay to the ${\text{X}}^{2}\Pi_{3/2}$ rather than ${\text{X}}^{2}\Pi_{1/2}$ according to the selection rules. The ${\text{A}}^{2}\Delta_{5/2}$→${\text{X}}^{2}\Pi_{3/2}$ transition can ensure a closed-loop cooling cycle, while the other two transitions can not. The three-laser cooling scheme proposed in the following using the ${\text{A}}^{2}\Delta_{5/2}$→${\text{X}}^{2}\Pi_{3/2}$ transition is more feasible than the one proposed by Zhang et al. (2018) without including the SOC effects. More importantly, they did not consider the effect of electronic state crossing, and proposed a laser cooling cycle for SiH involving the ν′ = 1 vibrational level of the A^2^Δ state, which would predissociation before the radiative transition and can not be used to establish laser cooling cycles.

**Figure 8:**
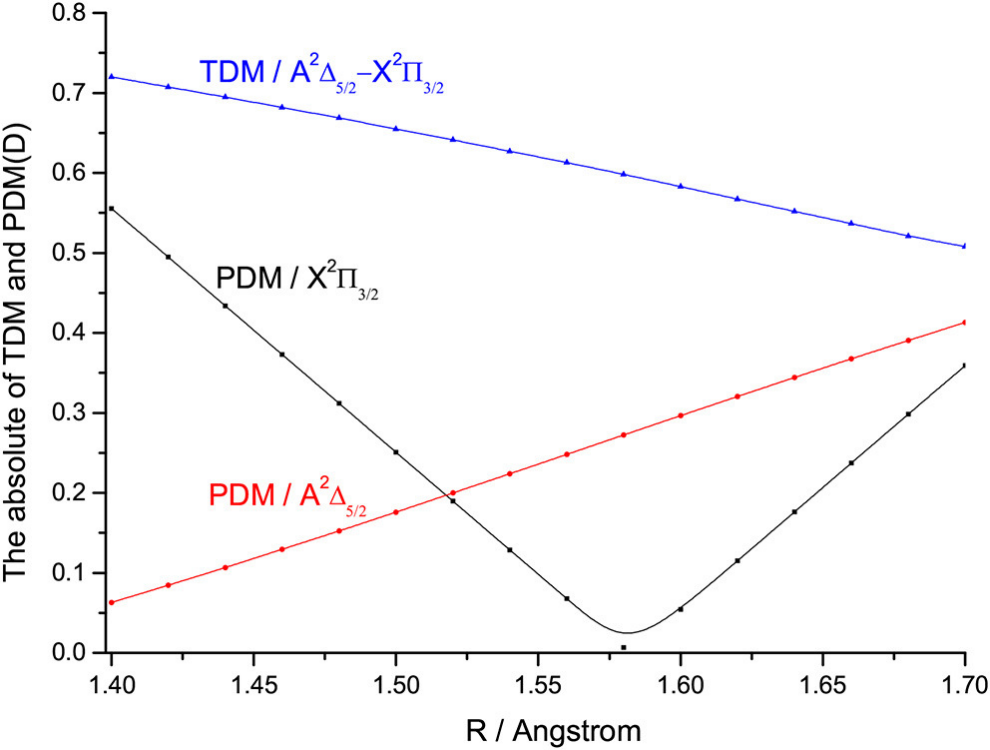
The permanent dipole moments (PDMs) and transition dipole moments (TDMs) for the ${\text{X}}^{2}\Pi_{3/2}$ and ${\text{A}}^{2}\Delta_{5/2}$ states of SiH at the icMRCI+Q level.

**Figure 9:**
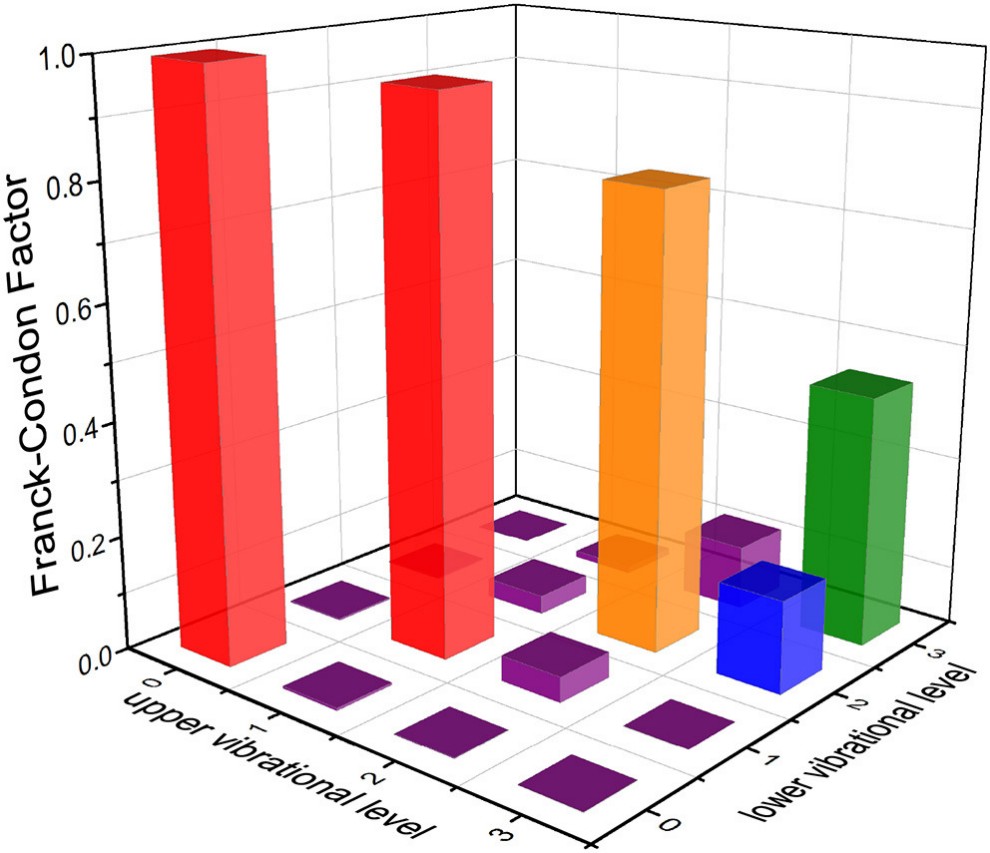
Franck-Condon factors of the ${\text{A}}^{2}\Delta_{5/2}$(ν′ ≤ 3)→${\text{X}}^{2}\Pi_{3/2}$(ν ≤ 3) transition for SiH, calculated at the icMRCI+Q level.

The TDMs of SiH for the ${\text{A}}^{2}\Delta_{5/2}$→${\text{X}}^{2}\Pi_{3/2}$ transition at the icMRCI+Q level are represented in Figure 8. The TDMs of SiH is 0.6157 D at *R*_*e*_. The FCFs values of the ${\text{A}}^{2}\Delta_{5/2}$→${\text{X}}^{2}\Pi_{3/2}$ transition for SiH are computed and shown in Figure 9. As seen, the Δν = 0 transitions are significantly larger than those for the off-diagonal terms. The present value of *f*_00_ (0.9949) is so large that the spontaneous decays to ν = 1,2 vibrational levels in the ${\text{X}}^{2}\Pi_{3/2}$ are significantly restrained. Additionally, the relative strengths of the photon loss pathways are more directly related to the vibrational branching ratios than the FCFs in the laser cooling cycle (Lane, 2015). Furthermore, we calculate the Einstein A coefficients $A_{\nu^{\prime }\nu }$ and vibrational branching ratios $R_{\nu^{\prime }\nu }$ of the ${\text{A}}^{2}\Delta_{5/2}$→${\text{X}}^{2}\Pi_{3/2}$ transition for SiH. The $A_{\nu^{\prime }\nu }$ and $R_{\nu^{\prime }\nu }$ of the ${\text{A}}^{2}\Delta_{5/2}$→${\text{X}}^{2}\Pi_{3/2}$ transition are listed in Table 4. As seen in Table 4, a very large *A*_00_ (1.73 × 10^6^ s^−1^) and very low scattering probabilities into off-diagonal bands (*R*_01_ = 3.61 × 10^−3^, *R*_02_ = 9.83 × 10^−4^, *R*_03_ = 9.20 × 10^−8^) of SiH contribute to a desirable condition for rapid and efficient laser cooling. It should be noted that *R*_00_ (0.9954) is slightly larger than *f*_00_ (0.9949), indicating that the probability of spontaneous decay to the ${\text{X}}^{2}\Pi_{3/2}$(ν = 0) increases when the variations in transition wavelength are taken into account.

**Table 4:** Calculated Einstein A coefficients $A_{\nu^{\prime }\nu }$ and vibrational branching ratio $R_{\nu^{\prime }\nu }$ of the ${\text{A}}^{2}\Delta_{5/2}$ (ν′) →${\text{X}}^{2}\Pi_{3/2}\left(\nu \right)$ transition for SiH.

	**ν^**′**^=** **0**		**ν^**′**^=** **1**		**ν^**′**^=** **2**		**ν^**′**^=** **3**	
	**$R_{\nu^{\prime }\nu }$**	**$R_{\nu^{\prime }\nu }$**	**$A_{\nu^{\prime }\nu }$**	**$R_{\nu^{\prime }\nu }$**	**$A_{\nu^{\prime }\nu }$**	**$R_{\nu^{\prime }\nu }$**	**$A_{\nu^{\prime }\nu }$**	**$R_{\nu^{\prime }\nu }$**
ν = 0	1.73 × 10^6^	0.9954	5.83 × 10^4^	4.13 × 10^−3^	3.50	2.83 × 10^−6^	5.49 × 10^2^	5.61 × 10^−4^
ν = 1	6.28 × 10^3^	3.61 × 10^−3^	1.40 × 10^6^	0.9954	2.06 × 10^5^	0.166	8.76 × 10^3^	8.95 × 10^−3^
ν = 2	1.71 × 10^3^	9.83 × 10^−4^	3.55 × 10^2^	2.52 × 10^−5^	1.00 × 10^6^	0.807	3.99 × 10^5^	0.41
ν = 3	0.16	9.20 × 10^−8^	6.43 × 10^3^	4.56 × 10^−4^	1.59 × 10^4^	1.28 × 10^−2^	4.83 × 10^5^	0.49

The vibrational branching ratios $R_{\nu^{\prime }\nu }$ is determined by the following expression:

(3) $$\begin{array}{l} R_{\nu^{\prime }\nu }=A_{\nu^{\prime }\nu }/\underset{\nu }{\sum }A_{\nu^{\prime }\nu } \end{array}$$

Moreover, the Doppler temperature (*T*_*Doppler*_) is the achievable minimum temperature of translational cooling with the Doppler method, it is obtained by the following expression:

(4) $$\begin{array}{l} T_{Doppler}=h/\left(4k_{B}\pi \tau \right), \end{array}$$

where *k*_*B*_ and *h* are Boltzmann′s constant and Planck′s constant, respectively (You et al., 2016). The radiative lifetime (τ) for the ${\text{A}}^{2}\Delta_{5/2}$(ν′ = 0) of SiH is 575 ns and *T*_*Doppler*_ is 6.65 μK. Additionally, the recoil temperature (*T*_*recoil*_) is calculated by the following expression:

(5) $$\begin{array}{l} T_{recoil}=h^{2}/\left(mk_{B}\lambda^{2}\right), \end{array}$$

and the obtained *T*_*recoil*_ for the ${\text{A}}^{2}\Delta_{5/2}(\nu^{\prime }=0)\to {\text{X}}^{2}\Pi_{3/2}(\nu =0)$ transition of SiH is 3.89 μK.

**Figure 10:**
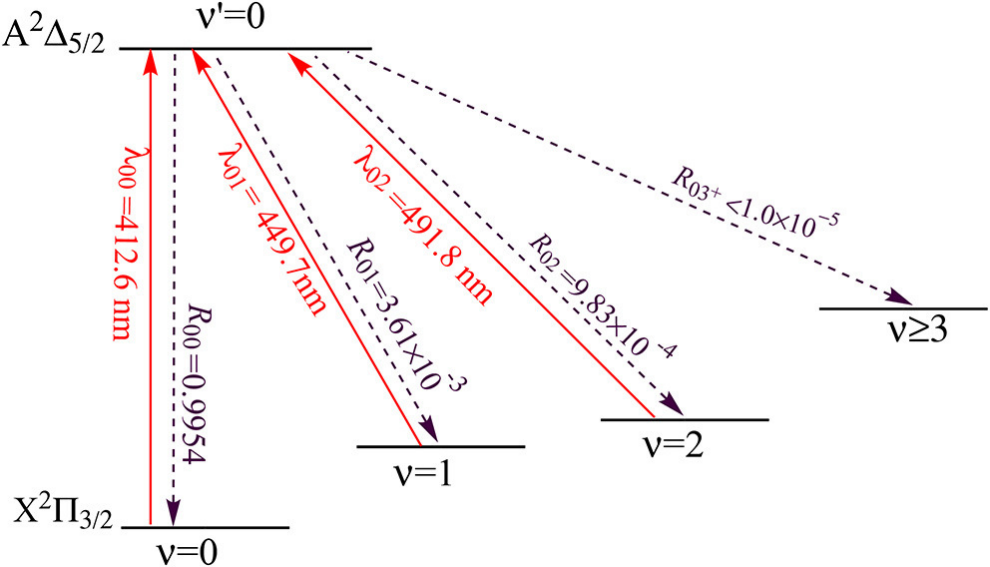
Proposed three-laser cooling scheme for SiH using the ${\text{A}}^{2}\Delta_{5/2}$→${\text{X}}^{2}\Pi_{3/2}$ transitions. Solid arrows indicate laser-driven transitions at certain wavelengths $\lambda_{\nu^{\prime }\nu }$. Dashed arrows indicate spontaneous decays from the ${\text{A}}^{2}\Delta_{5/2}(\nu^{\prime }=0)$ state with the calculated $R_{\nu^{\prime }\nu }$.

The proposed scheme to facilitate the laser cooling of SiH is shown in Figure 10. The large *R*_00_ (0.9954) of SiH indicates that the ${\text{A}}^{2}\Delta_{5/2}(\nu^{\prime }=0)\to {\text{X}}^{2}\Pi_{3/2}(\nu =0)$ transition has the largest probabilities. A desirable laser cooling cycle needs to solve the vibrational branching loss. Therefore, the off-diagonal vibrational branching ratios $R_{\nu^{\prime }\nu }$ of SiH are calculated, and the probabilities of decay from the ${\text{A}}^{2}\Delta_{5/2}$(ν ′ = 0) to the ${\text{X}}^{2}\Pi_{3/2}$(ν = 1, 2) are firstly obtained (*R*_01_ = 3.61 × 10^−3^ and *R*_02_ = 9.83 × 10^−4^). Besides, the probabilities of the unwanted decay channels are also computed by using *R*_03+_+*R*_02_×*R*_13+_. The negligible value of 1.0 × 10^−5^ means that the present scheme will allow for at least 1.0 × 10^5^ photon absorption/emission cycles, which are sufficient enough to decelerate SiH in a cryogenic beam, in principle (Shuman et al., 2010). The laser cooling scheme takes the transition ${\text{A}}^{2}\Delta_{5/2}(\nu^{\prime }=0)\leftarrow {\text{X}}^{2}\Pi_{3/2}(\nu =0)$ as the main pump, ${\text{A}}^{2}\Delta_{5/2}(\nu^{\prime }=0)\leftarrow {\text{X}}^{2}\Pi_{3/2}(\nu =1)$ and ${\text{A}}^{2}\Delta_{5/2}(\nu^{\prime }=0)\leftarrow {\text{X}}^{2}\Pi_{3/2}(\nu =2)$ as the first and second vibrational repump, respectively. Accurate *T*_*e*_ is crucial for estimating the pump and repump wavelengths in laser cooling cycles, and our calculated *T*_*e*_ values, which are very close to experimental ones, give confidence in the subsequent study on laser cooling, especially, for SiH. The calculated value of wavelength λ_01_ should be larger than λ_00_, however, the wavelengths λ_01_ obtained by Zhang et al. (2018), is 376.88 nm, which is smaller than that of their main pump. In our laser-driven cycling, the calculated pump and repump wavelengths of λ_00_, λ_01_, and λ_02_ are 412.6, 449.7, and 491.8 nm, respectively. The required wavelengths are all in the range of 400–500 nm and can be produced with the frequency doubled semiconductor laser, which has been used for the laser cooling experiment of the strontium atom (Wang et al., 2009). The SiH molecules will stay in the laser cooling cycle until the decay to ν≥3 (${\text{X}}^{2}\Pi_{3/2}$).

### 3.4. Laser Cooling Scheme for CS

**Figure 11:**
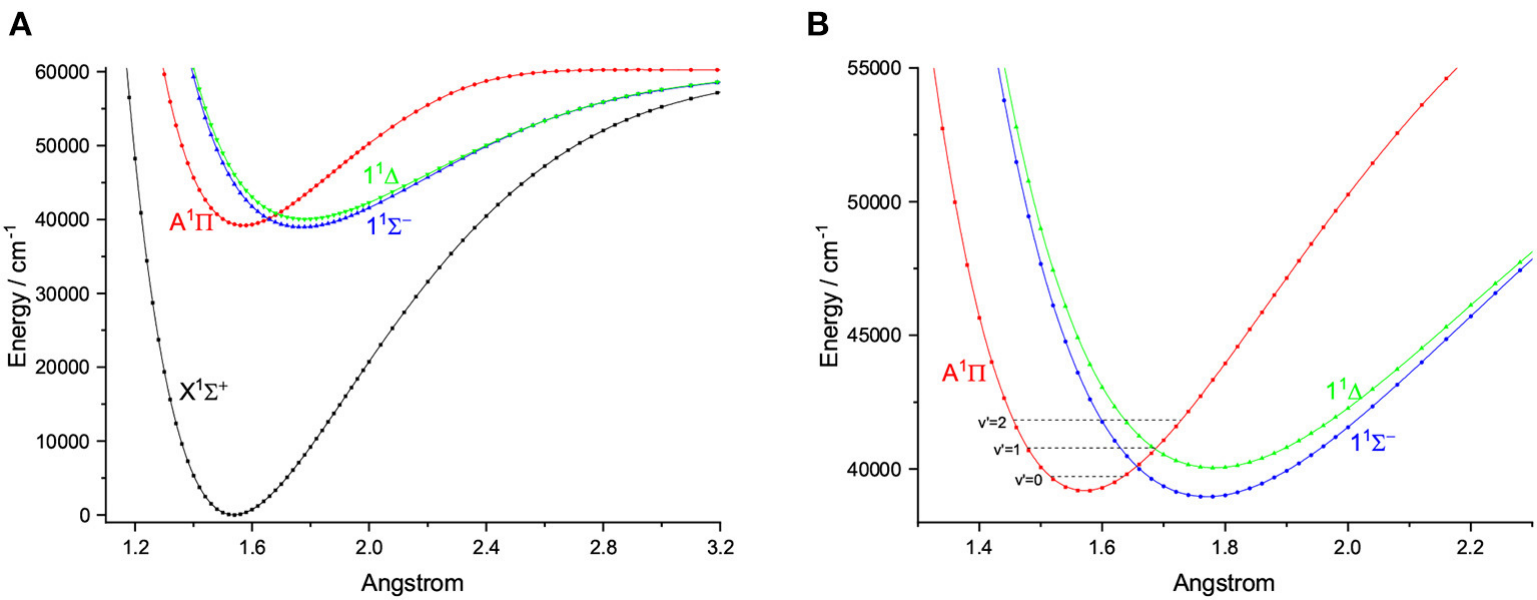
Potential energy curves of CS for the four Λ-S states at the icMRCI+Q level **(A)** and an amplified view of crossing regions of potential energy curves **(B)**.

We further find that CS is a promising candidate based on the criteria mentioned above. The PECs of CS, obtained at the icMRCI+Q level, is shown in Figure 11. Our calculated *R*_*e*_ and ω_*e*_ of the X^1^Σ^+^ of CS are 1.5380 Å and 1288.63 cm^−1^, respectively, which are in very good accordance with the corresponding experimental values (1.5349 Å and 1285.1 cm^−1^) (Huber and Herzberg, 1979). As for the A^2^Δ state of CS, our calculated *T*_*e*_ 39175.96 cm^−1^ is in agreement with the experimental value 38,904 cm^−1^ (Huber and Herzberg, 1979). The calculated vibrational branching ratio *R*_00_ is 0.885, which is highly diagonal.

**Figure 12:**
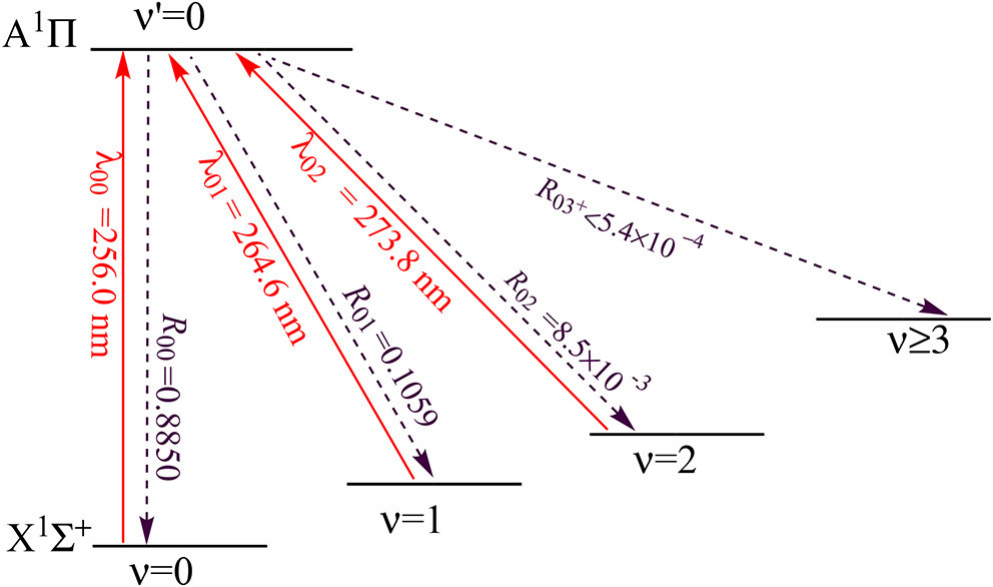
Proposed three-laser cooling scheme for CS using the A^1^Π→X^1^Σ^+^ transitions. Solid arrows indicate laser-driven transitions at certain wavelengths $\lambda_{\nu^{\prime }\nu }$. Dashed arrows indicate spontaneous decays from the A^1^Π(ν′ = 0) state with the calculated $R_{\nu^{\prime }\nu }$.

As shown in Figure 11, the PEC of A^1^Π crosses with those of two other electronic states, however, from an amplified view of the crossing regions we see that, the two crossing points are higher than the ν′ = 0 vibrational level of the A^1^Π state. So this kind of crossing would not affect laser cooling cycles using the A^1^Π(ν′ = 0)→X^1^Σ^+^(ν <3) transition. A suitable laser cooling scheme using the A^1^Π→X^1^Σ^+^ transition of CS is proposed and shown in Figure 12. The scheme takes the transition A^1^Π(ν′ = 0)→X^1^Σ^+^(ν = 0) as the main pump, A^1^Π(ν′ = 0)→X^1^Σ^+^(ν = 1) and A^1^Π(ν′ = 0)→X^1^Σ^+^(ν = 2) as the first and second vibrational repump, respectively. The radiative lifetime of the A^1^Π state is 70 ns, whereas the T_*Doppler*_ and T_*recoil*_ of the A^1^Π→X^1^Σ^+^ transition are 54.61 and 6.64 μK, respectively. The large *R*_00_, short radiative lifetime and ultracold temperatures suggest CS as a promising candidate for rapid and efficient laser cooling.

## 4. Conclusions

The fourth criterion for molecular laser cooling is proposed in this work, that is, there is no electronic-state crossing, or the crossing point is high enough in energy. Its importance is demonstrated by investigating the laser cooling feasibility of group IVA hydrides and carbon monosulfide.

*Ab initio* and dynamical calculations are performed, and the calculated spectroscopic constants are in very good agreement with the available experimental data. We find that the locations of crossing point between the B^2^Σ^−^ and A^2^Δ states have the tendency of moving downwards from CH to SnH relative to the bottom of the corresponding A^2^Δ potential, and this would lead to failure of cooling GeH and SnH. The potential wells of the A^2^Δ state of group IVA hydrides become shallower and shallower from CH to PbH, with the Franck-Condon factor decreasing, which results in a very small Franck-Condon factor for PbH. It is clear that PbH is not a suitable candidate for laser cooling either. We further propose a practical and efficient laser-cooling scheme for SiH using the ${\text{A}}^{2}\Delta_{5/2}$→${\text{X}}^{2}\Pi_{3/2}$ transition. The calculated excitation energy is 24299.20 cm^−1^, which is in perfect agreement with the experimental data (24300.4 cm^−1^). This allows us accurately estimate the pump and repump wavelengths in laser cooling cycles, which is shown to vary from 400 to 500 nm and are easily accessible in experiment. The Doppler temperature and recoil temperature of SiH for the ${\text{A}}^{2}\Delta_{5/2}$→${\text{X}}^{2}\Pi_{3/2}$ transition are 6.65 and 3.89 μK, respectively. The computed radiative lifetime is 575 ns, and the vibrational branching ratio is highly diagonally distributed with the *R*_00_ being 0.9954. Furthermore, we performed additional calculations, and find that, the carbon monosulfide (CS) is a promising candidate which meets the four criteria, and we further propose a suitable laser cooling scheme. We hope that this work will be helpful in searching for promising candidates for producing ultracold molecules.

## Data Availability Statement

All datasets generated for this study are available within the article and from the corresponding author on request.

## Author Contributions

DL carried out the MRCI calculations. DL, MF, HM, and WB analyzed the data and interpreted the results. ZD and CC provided computing assistance. DL, HM, and WB developed the theoretical scheme and wrote the paper. WB supervised the research and proposed the fourth criterion for molecular laser cooling.

### Conflict of Interest

The authors declare that the research was conducted in the absence of any commercial or financial relationships that could be construed as a potential conflict of interest.
