# Supplementary material for: A Theoretical Study on Laser Cooling Feasibility of Group IVA Hydrides XH (X = Si, Ge, Sn, and Pb): The Role of Electronic State Crossing
Source: Front Chem. 2020 Jan 28;8:20. doi: 10.3389/fchem.2020.00020 (PMC6997332; doi:10.3389/fchem.2020.00020)
Supplement: Supplementary file 1 [file Data_Sheet_1.PDF]

Supplementary Material

1 SUPPLEMENTARY TABLES AND FIGURES

1.1 Tables

Table S1. Spectroscopic constants of the $A^2\Delta$ and $X^2\Pi$ states for SnH.

State	Method	T_e (cm^{-1})	R_e (Å)	ω_e (cm^{-1})	B_e (cm^{-1})
$X^2\Pi$	This work		1.7706	1737.91	5.3811
	Expt.[a]		1.78146		
$A^2\Delta$	This work	24268.34	1.8200	1224.17	5.1339
	Expt.[a]		1.8546		

[a] Ref. (Huber and Herzberg, 1979).

Table S2. Calculated Einstein A coefficients $A_{\nu'\nu}$ and vibrational branching ratio $R_{\nu'\nu}$ of the $A^2\Delta$ (ν') $\rightarrow X^2\Pi(\nu)$ transition for GeH.

	ν'=0		ν'=1		ν'=2		ν'=3	
	$A_{\nu'\nu}$	$R_{\nu'\nu}$	$A_{\nu'\nu}$	$R_{\nu'\nu}$	$A_{\nu'\nu}$	$R_{\nu'\nu}$	$A_{\nu'\nu}$	$R_{\nu'\nu}$
ν=0	2.54×10^{6}	9.95×10^{-1}	3.93×10^{5}	2.10×10^{-1}	3.68×10^{4}	2.90×10^{-2}	2.43×10^{3}	2.65×10^{-3}
ν=1	6.73×10^{3}	2.64×10^{-3}	1.40×10^{6}	7.51×10^{-1}	8.01×10^{5}	6.32×10^{-1}	2.65×10^{5}	3.25×10^{-1}
ν=2	6.27×10^{3}	2.46×10^{-3}	4.82×10^{4}	2.59×10^{-2}	3.32×10^{5}	2.62×10^{-1}	5.11×10^{5}	6.47×10^{-1}
ν=3	2.00	7.85×10^{-7}	2.14×10^{4}	1.15×10^{-2}	5.57×10^{4}	4.40×10^{-2}	3.50×10^{2}	6.22×10^{-4}

1.2 Figures

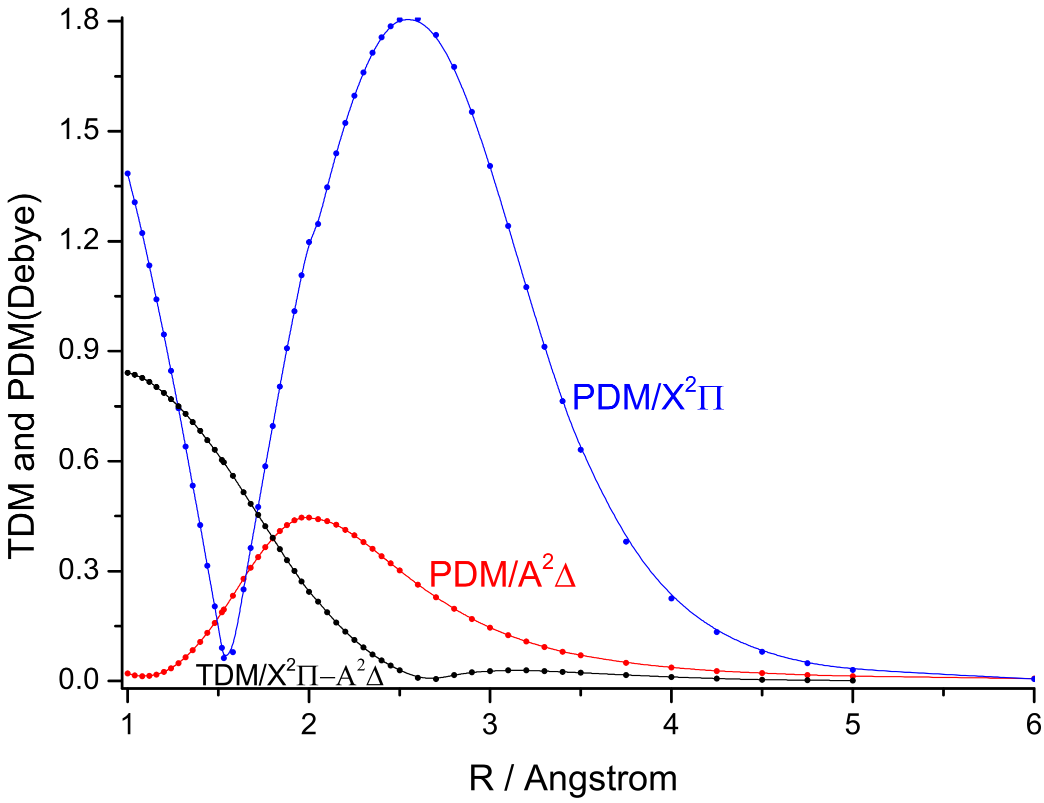

Figure S1. The permanent dipole moments (PDMs) and transition dipole moments (TDMs) for the $X^2\Pi$ and $A^2\Delta$ states for GeH at the icMRCI+Q level.

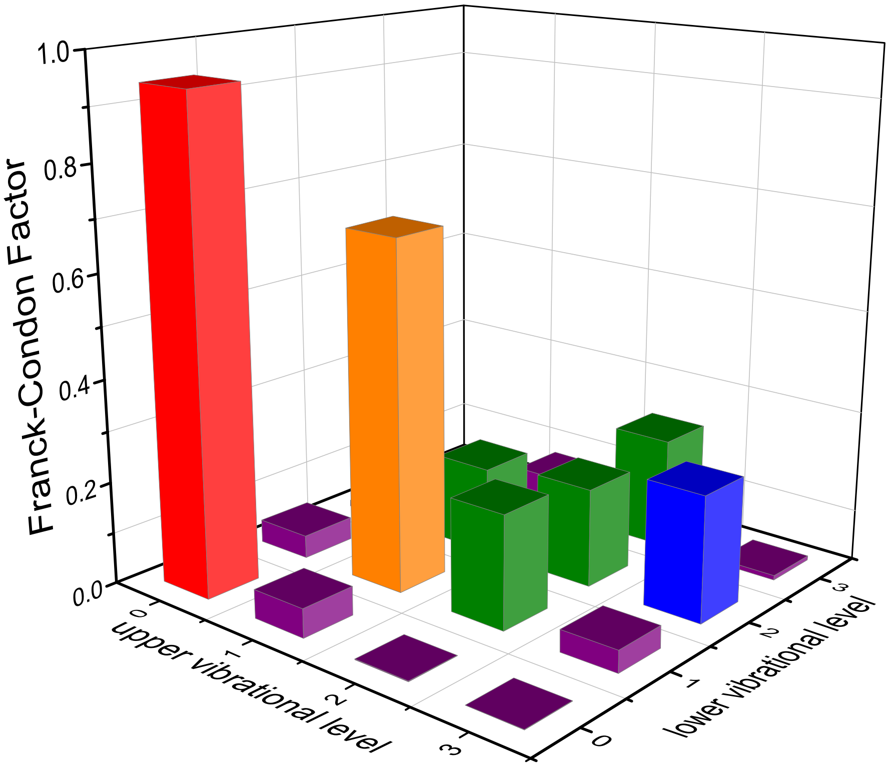

Figure S2. Franck-Condon factors of the $A^2\Delta(\nu' \leq 3) \rightarrow X^2\Pi(\nu \leq 3)$ transition for GeH, calculated at the icMRCI+Q level.

REFERENCES

Huber, K. P. and Herzberg, G. (1979). *Molecular spectra and molecular structure IV: Constants of diatomic molecules.* (Van Nostrand Reinhold: New York)
